# The New York Institute of Stomatology

**Published:** 1897-07

**Authors:** 

**Affiliations:** The New York Institute of Stomatology


### THE NEW YORK INSTITUTE OF STOMATOLOGY.

A regular meeting of the Institute was held Tuesday evening, March 2, 1897, at the residence of Dr. J. Morgan Howe, No. 58 West Forty-seventh Street, the President, Dr. Geo. S. Allan, in the chair.

The minutes of the previous meeting were read and approved.

COMMUNICATIONS ON THEORY AND PRACTICE.

Dr. James G. Palmer read an article on "Defective Articulation," etc., by Dr. J. G. Brigiotti, of the École Odontotechnique, of Paris. (For Dr. Brigiotti's paper, see page 439.)

*Dr. S. E. Davenport.*—Dr. Brigiotti, it seems to me, should be commended for his very successful use of photography in illustration of the points of his paper.

I have many times thought how much better the changes which are brought about by prosthetic appliances could be shown if we had, as the patent medicine men are always careful to have, the pictures of the patient "before and after." If we had a particle of doubt regarding the advisability of Dr. Brigiotti following the plan which he has so concisely explained, the doubt would be dispelled by one glance at these excellent photographs, for there is noticeable a very marked and advantageous change in the appearance of the patient's features.

*Dr. Benjamin Lord.*—There seems to be a great deal of interest in the report just presented, the casts and photographs in illustration being of special value, and we can but feel greatly indebted to the friend who has thus contributed to our edification this evening. Accordingly, I move a most hearty vote of thanks to Dr. Brigiotti for his trouble and interest in our behalf.

The motion was carried.

*Dr. F. Milton Smith.*—About a year ago Dr. Kirk read a paper before the First District Dental Society on "Infantile Scorbutus." My attention was especially attracted to it at the time, as I had never seen a case in practice. Before two months had passed a lady patient came to me to ask if I could give her any light in regard to her baby's teeth and gums. The child had the two central incisors, and the gum was so much inflammed as to almost cover them, and was very black in color. The baby was thirteen months old. I found upon questioning the mother that the child had every symptom of infantile scorbutus. She had been able to walk, but had lost the use of her limbs. The physicians had treated the child for rheumatism, and one of them suggested that the child had dental paralysis and several other things. I suggested that the child be fed upon beef-juice and bread, also bread and milk and orange-juice. In the course of three weeks, upon this change of diet (the child having had almost exclusively malted milk from its birth), the little one was almost well again. That was about ten months ago.

About six weeks since a gentleman came to my office and told me about the condition his child had been in during the summer. She had been very much run down, and seemed to become pale without any apparent loss of appetite, her digestion was good, and yet the little one seemed to fade away. The child had only the two central incisors at that time, although it was about thirteen months old. It had the same condition of the gums as the other case. I asked if the child walked, and he said yes, but it had lost the use of its limbs, and seemed to suffer much pain when it was touched. He said the physician suspected that there was some trouble with the food, and so changed it, and the child began to improve immediately. Its food had been Carnrick's food almost exclusively from birth to the best of my recollection. The physician did not diagnose the case as infantile scorbutus, but I think there is no question about it. He ordered a change of food, and gave the child beef-tea, cream, cod-liver oil, etc., and it seemed to improve at once. Physicians seem to overlook the cases very often for some reason or other. I believe, up to about a year ago, there were only about forty cases reported by physicians in this country.

*Dr. C. B. Parker.*—Dr. Smith has repeated an experience I had in my own family only about a year ago. My physician wished to bring my child over here to a specialist, and I told him I thought if the food of the child were changed and it had some fruit that the conditions would be changed. The little one lost the use of her limbs for about a month; at the end of the month she was so she could be up on her feet again, but she did not walk for about three months. She was brought up on malted milk.

Dr. Brockway then read a paper by Dr. Louis Jack, of Philadelphia, entitled "Treatment of Devitalized Teeth."

(For Dr. Jack's paper, see page 370.)

*The President.*—It is well for us to have so complete a statement from so distinguished an operator as Dr. Jack as to his method of treating root-canals in all their various stages. I hope there will be a free and full discussion of the paper which is now before you.

### DISCUSSION.

*Dr. J. Morgan Howe.*—I am interested in Dr. Jack's use of aristol. I would like to ask if there is any one present who uses it?

*Dr. Babcock.*—I have used aristol in root-canals a little over two years, in combination with oil of cinnamon or oil of gaultheria and carbolic acid, and have had great success with it. I always try to get direct access to the cavity, believing in thorough mechanical cleansing first, then following with antiseptics. About four years ago a gentleman came to me complaining of the right upper first bicuspid, which had been treated and dressed for several months. I felt that unless there was some abnormality in that root there appeared to be no reason for the continuation of the trouble. I found there had been an opening made only large enough to admit a broach, so I opened the tooth thoroughly and found that there were two canals, as usual. One was most beautifully filled; the other had putrescent contents, and I did not wonder the trouble was going on. I merely mention this believing thorough access should always be had for mechanical cleansing, even though the tooth may apparently be weakened thereby. A tooth that behaves itself, even though more than half composed of filling, is far preferable to one that is always giving trouble, even though it be nearly sound.

*Dr. Brockway.*—I quite agree with Dr. Babcock as to the importance of mechanically cleansing a root, and I am glad Dr. Jack has put so much stress upon the importance of access to root-canals as the first step. The matter of treating pulpless teeth used to be a great bugbear, but with our present knowledge on the subject, I take these cases with very little apprehension. In fact, my assistant rather amused me, not long since, by wishing that some more "nerve cases" would come in. A few years ago to express such a wish would have seemed to be inviting trouble. In the treatment of roots with putrescent pulps my great reliance for the past three or four years has been on Dr. Schrier's preparation of kalium natrium. I know, however, that many are not using it as I am. I have found it extremely useful, shortening and simplifying the operation very much. By its use, with thorough attention to mechanical cleansing of the canals, I have escaped almost all untoward subsequent symptoms, such as we used to dread, and frequently had occur. As to filling root-canals where the root is a simple one and accessible, like a cuspid, incisor, or the palatal root of an upper molar, I do not know of any better method than that practised by Dr. Richmond for a number of years, and that is, filling the root with a peg of wood whittled out to fit it and dipped in the root-preparation, which I think Dr. Lord brought to our notice some years ago. I do not know that I have ever had trouble from a root filled in that manner, and I have filled hundreds of them. Within the past year I have used, instead of that preparation, the article introduced by Dr. White, of Silver City, N. M., called balsamo del deserto. It is a wax-like product of some insect, I fancy. It is antiseptic, insoluble, remains soft, and seems to me to be admirably adapted for the purpose of completely sealing the apical foramen of a root, and the entrance to the tubuli in the dentine.

*Dr. Babcock.*—I would like to say a word in regard to the use of aristol and the essential oils. This combination has such a tendency to discolor that if used in the front of the mouth it would not do to fill the root too full. I use a gutta-percha cone, forcing it up after saturating the canal with the fluid, and then use oxychloride of zinc over that. I found if I filled the entire root with the gutta-percha, it would be apt to discolor the tooth.

*The President.*—Aristol and oil of gaultheria I have used, as mentioned by Dr. Jack, for several years, with a great deal of satisfaction. I would like to call attention to the use Dr. Jack makes of permanganate of potasssium for putrescent canals. I know of no more effective method of destroying germs when a tooth of that description is opened and a piece of bibulous paper used to absorb the liquid contents of the pulp-chamber than to place a crystal of permanganate of potassium at the entrance to the canal, and allow it to remain there for ten or fifteen minutes, the tooth then being syringed out with tepid water, and closed in such a way as not to force anything through the apical foramen. It is almost impossible for a root treated in that manner to retain germs of any character that will do harm. I heard of this through Dr. Jack, and my first question, when he told me of it, was about the discoloration. He replied, as I have since found in practice to be true, that the danger was very slight, especially if a little oxalic acid is used. Although I have used this method repeatedly in the last year, I have yet to see a case where the discoloration amounted to anything. The beneficial results have been most marked and satisfactory.

*Dr. Gage.*—May I ask Dr. Allan what he does with roots where there seem to be no contents,—where they seem to be perfectly sweet, and there is no odor whatever? Does he fill those immediately?

*The President.*—The mere fact that there is no odor does not indicate definitely that there are no pus-germs, or germs that will induce inflammation, and while the treatment indicated may not be so positive and decisive as in cases where there is an offensive odor, it must be applied with the utmost care. In those cases I use aristol and oil of gaultheria. I have also used two and one-half to five-per-cent. formalin solution.

*Dr. Davenport.*—In my opinion, Dr. Jack should be thanked, not only for his very complete description of most excellent methods of treatment, but for his thorough honesty,—although that we would naturally expect from him. Many of us have been so accustomed to the descriptions of essayists, where everything is claimed, even the perfect mechanical cleansing of the buccal roots of upper molars and the anterior roots of lower molars, that I am sure we all notice Dr. Jack's confession and are refreshed thereby.

*The President.*—One more point I would mention. The use of germicides in the future will be largely determined by one fact,—whether they are organic or inorganic in their origin. I do not believe there is any organic germicide, other things being equal, that is as reliable as an inorganic one. Oil of gaultheria, or oil of cinnamon, on a pallet of cotton left in a cavity is sure, sooner or later, to become food for germs, if there are germs there. It is a wise thing, therefore, where one has a choice, to choose the inorganic germicide rather than the organic.

*Dr. Geo. A. Maxfield.*—This subject is a very broad one. I have just closed the third lecture on this subject at the dental school to-day, and have hardly covered the ground. Dr. Jack divides the subject into three heads, but we all know that these heads can be subdivided almost innumerably. I can heartily commend almost everything in Dr. Jack's paper, yet I do not use aristol. I have tried at various times the different methods advised for the treatment of pulpless teeth, but fail to find a better method than the one I have used for the past ten years. I seldom place the dam on a tooth either where I want to open into the pulp-chamber or treat a pulpless tooth. To me it is in the way. My first precaution is to get the mouth into an antiseptic condition, and that I do by using a strong solution of hydronaphthol as a mouth-wash; then I see that the cavity is mechanically cleansed. In opening the pulp-chamber I cover the parts and have my drill covered with a saturated solution of iodoform in eucalyptol. I use iodoform a great deal in treating pulpless teeth, and I have yet to learn of any remedy that will do what iodoform does. We need something besides a germicide and an antiseptic. We must destroy the germs of course, but we must also destroy the infection,—the ptomaines,—and nothing will do this, in my opinion, like iodoform. In regard to making the opening through the gums, Dr. Jack uses what to me seems an exaggerated term. I never hear this word, alveolotomy, but what immediately the word ovariotomy flashes through my mind, and with it the comparison of the two operations. The attempt to magnify the simple operation of piercing the alveolus by calling it alveolotomy is hardly warranted. One point I particularly emphasized in my lecture to-day was that great care must be exercised in making the opening through the alveolus to reach the end of the root of the second inferior bicuspids. Often the roots of these teeth are very long, and there is danger of penetrating the mental foramen. A case of this kind came under my observation a few years ago, in which paralysis of the lip and chin resulted, lasting several weeks. I use the peroxide of hydrogen more as a mechanical cleanser than as an antiseptic. In its effervescence, it throws out the *débris* from the canals. I use Dr. Callahan's method, the acid treatment for opening the canals. Since adopting this method I have many times been surprised, when treating the superior molars, to find four canals. After opening into the canals I prefer to use the chemically pure hydrochloric acid, full strength, instead of sulphuric acid, and use chlorinated soda to neutralize the acid. In pericemental inflammation I have used for several months, with excellent results, the cataphoric application of the compound tincture of iodine. To illustrate this: A young man while attending Harvard College had one of the lower molars treated and filled, but the filling had to be removed the next day. Three attempts were subsequently made to fill this tooth without success. When he came to me a few weeks ago, the cavity and canals had remained open for over two years. After thoroughly washing out the canals I placed a dressing of a five-per-cent. solution of formalin in the canals and sealed the cavity with gutta-percha, giving instructions to return if any trouble occurred. In the afternoon he returned and said he would be forced to have the filling removed as the tooth was beginning to trouble him. I gave a cataphoric application of the tincture of iodine for ten minutes, and there has been no trouble with this tooth since.

*Dr. Evans.*—Dr. Maxfield has made a statement that involves a scientific question. He said iodoform destroys the ptomaines and aristol will not. I would like to ask for his reasons for the statement. The teeth in which we want to destroy ptomaines are those in which we find a putrescent pulp or a septic condition present. In such cases we want an agent that will do more than destroy the ptomaines. We want one that additionally will exert a chemical action on sulphuretted hydrogen and other gases present and aid in eleminating them. By other gases present, I mean those ethereal gases, stinking gases, described by Vaughan and other writers who have made a study of putrefaction.

*Dr. Maxfield.*—I am not a scientific chemist or bacteriologist to go into that. I watch and study everything that I can find in regard to the subject, and I have yet to learn of prominent experimenters who make the statement that aristol will destroy the ptomaines. It may have been made, but I have not seen it.

*The President.*—What foundation has Dr. Maxfield for his statement that iodoform does destroy the ptomaines? Why is destroying the ptomaines of more importance than destroying the germs?

*Dr. Maxfield.*—Those who are acquainted with the history of iodoform will remember that this fact was very strongly brought out when it was shown that the bacteria were able to live in the iodoform powder; the surgeons began to wonder why they had such wonderful success with iodoform, and many theories were advanced. Finally, while every one admitted that it would not destroy bacteria, they found by inserting iodoform into the wound and then inserting bacteria, no evil results followed. By inserting the bacteria first and then the iodoform, they had bad results; then they isolated the ptomaines from the bacteria, and by introducing the ptomaines and then the iodoform there were no evil results. As it was impossible to isolate the bacteria from the ptomaines, they could not prove that it had no effect on the bacteria, if there were no ptomaines.

*Dr. Evans.*—That does not answer the question. I asked why iodoform destroys the ptomaines and aristol does not. That is the point I desire to be informed upon. It is an important one to us as dentists to know whether we should use aristol, a compound of thymol and iodine, which has an agreeable odor, or use iodoform, the odor of which patients can detect the moment they enter the front door. I cannot tolerate disagreeable odors of drugs in my office. I use a saturated solution of aristol with the essential oils. If I gave my record to this society for the last five years with the use of aristol it would scarcely be believed. The benefit we get from the use of either of these substances, as I understand it, is that the free iodine is eliminated. In the most grave operations in the Post-Graduate Medical School they use aristol in place of iodoform. With aristol we get all the effects derived from such a substance as iodoform, and there is not the disagreeable odor.

*Dr. Gage.*—May I ask Dr. Maxfield whether he has ever had a tooth discolor yellow from the use of iodoform?

*Dr. Maxfield.*—Never.

*Dr. Gage.*—I can show one. The only thing that has been in that tooth is iodoform and eucalyptus. There is a yellow color, and I would like to get it out.

*Dr. Smith.*—I gather from Dr. Jack's paper that it is the exception rather than the rule for him to fill roots of teeth without continued treatment. I would like to ask whether that is the accepted method with the gentlemen here present. I confess it is not mine. It is the exception rather than the rule for me to treat repeatedly, sitting after sitting.

*Dr. Babcock.*—That thought occurred to me when Dr. Brockway was reading the article. When I first began to practise I did treat teeth time and time again. I afterwards lessened the number of treatments, and now my custom is at the first visit to use hydrogen dioxide to cleanse the canal, then drying as thoroughly as possible, using strong alcohol to evaporate any moisture present, and sealing the cavity after introducing the preparations I spoke of. At the next visit I almost invariably fill the tooth permanently, and have no more trouble than by treating for six months.

Dr. J. Morgan Howe then read a paper entitled, "Some Points in the Antiseptic Treatment of Root-Canals."

(For Dr. Howe's paper, see page 364.)

DISCUSSION.

*The President.*—Dr. Howe's paper is now before the Institute for discussion. Does the chair understand Dr. Howe that he would state it as a fact that there is no albumen present?

*Dr. Howe.*—I suppose it to be a fact, as I have stated, and I have tried to coagulate putrid matter, but have not succeeded in doing so. I will not say that it cannot be coagulated, but I have not succeeded in doing so.

*Dr. Littig.*—Dr. Howe does not believe that there is any albumen in there, as such?

*Dr. Howe.*—No; the albumen has been disorganized; it ceases to be albumen, because it has been disintegrated, or split up.

*Dr. Maxfield.*—With the nitrate of silver, what effect is obtained? Is it coagulation or is it something else?

*Dr. Howe.*—I am not prepared to say. I have not tried it on putrescent matter.

*Dr. Maxfield.*—I would like to thank the Executive Committee for presenting two such valuable papers, and I feel that I have been most fortunate to have had the privilege of hearing them. I am very much interested in the application of nitrate of silver in the treatment of pulpless teeth, and I would like to ask Dr. Howe if he can give us anything more than he gave in the paper. Is there any evil results to the pericemental tissue from the transfusion of the nitrate of silver through the root of the tooth?

*Dr. Howe.*—I know practically nothing about silver nitrate in the roots of teeth. My favorable opinion of it is based upon what I know of its action in uniting with organic matter elsewhere. The demonstration that Dr. Bethel made of the possibility of forcing the salt into root-canals in such a way as to completely fill them, as those specimens showed that were sent here for exhibition, seemed to me to suggest an entirely new way of filling canals. I have no doubt that the tissues beyond the apex of the root will be perfectly safe when the proper amount of galvanic current for use in such cases has been determined. I should suppose the best results would follow, when a certain amount of organic matter remained in the canal treated with silver nitrate, for it is not the filling of the canal with the salt that is to be desired, but with the compound it forms with organic matter. And I should suppose that periapical tissues would be most likely to be injured, when the root-canal had been previously cleansed.

*Dr. Littig.*—Would not the precipitation of the nitrate of silver take place in contact with albumen, if it passed out into normal tissue and so render it comparatively harmless?

*Dr. Howe.*—I should not call it precipitation. Silver nitrate is a caustic and is entirely capable of destroying tissue as such. It would as quickly convert the living as dead tissue into a new compound, and it might be possible to do harm with it. We must determine the amount of galvanic current proper to use for the purpose, I think, before we can be sure that we may not do harm, but the practical results obtained by those who have used it are of far greater value than any opinions formed without practice of the method.

*Dr. Babcock.*—The presence of any organic substance in aqueous solutions of nitrate of silver will generally precipitate the silver almost instantly, consequently, the nitrate of silver being carried into a tooth in the form of an aqueous solution would be precipitated, and there would be none to go through the foramen.

*Dr. McNaughton.*—Dr. Bethel said he could not force it through, no matter how long he tried.

*Dr. Babcock.*—Since he forced it into the tubuli, there is no reason to suppose that it could not be forced through the foramen.

*Dr. Maxfield.*—In regard to the use of chloride of zinc, some may remember the controversy between Drs. Harlan and Kirk. I had a little experience at that time, which Dr. Kirk used to prove his ground. Having some correspondence with him, and being interested in the controversy, I wrote to him that I had two cases that proved his theory. He answered at once, requesting me to give him the details, and my description appeared in the *Dental Cosmos*

under "Hints and Queries." In those two cases of lower molars, I had placed the dam on, and removed the pulp with cocaine. To coagulate the stump end of the pulp, which is necessary in those cases, I had used the chloride of zinc. This set up a severe inflammation in the pericementum, which took considerable time to allay. There was normal albumen and there must have been coagulation in the canal, yet it did not prevent the chloride of zinc from penetrating through to the pericemental tissue.

*Dr. Brockway.*—If any gentleman has ever undertaken to fill root-canals with the oxychloride of zinc, he probably has had some little experience with the effect of chloride of zinc when forced through the foramen. I remember I had two or three such cases, and the recollection of them is not very pleasant. Since then I have not used it for that purpose. This, of course, was many years ago.

Adjourned.

S. E. Davenport, D.D.S., M.D.S.,
*Editor The New York Institute of Stomatology.*

